# Modeling the acceptance of vegetarian diets to promote sustainable food systems

**DOI:** 10.1002/fsn3.4308

**Published:** 2024-08-09

**Authors:** Caroline Rajeh, Jean B. Hunter, David A. Levitsky, Myra Zeineddine, Samer A. Kharroubi, Ammar Olabi

**Affiliations:** ^1^ Department of Nutrition and Food Sciences, Faculty of Agricultural and Food Sciences American University of Beirut Beirut Lebanon; ^2^ Department of Biological and Environmental Engineering, College of Agricultural and Life Sciences Cornell University Ithaca New York USA; ^3^ Division of Nutritional Sciences, College of Human Ecology Cornell University Ithaca New York USA

**Keywords:** food consumption, meal acceptability, multiple imputation, sustainable food systems, vegetarian diet

## Abstract

With increasing concerns about health, animal welfare, and the environment, changes in dietary patterns are emerging, as evidenced by the gradual shift toward plant‐rich diets. To appropriately plan vegetarian meals with high consumer satisfaction that would help promote this dietary pattern and ultimately improve the sustainability of food systems, meal acceptability testing would be crucial. The present work aims to investigate the influence of individual food components' acceptability on the overall meal acceptability in vegetarian diets. Over four taste‐testing periods, 94 panelists of US nationality recruited from Cornell University staff (47 males and 47 females; 30–50 years of age) were asked to rate the acceptability of 41 vegetarian meals containing various food category items, including a main dish, side dish, appetizer, salad, soup, bread, dessert, beverage, and a control food. Each meal included six menu items to ensure the tested foods resembled a typical meal. Consequently, some meals lacked one or more of the nine total food categories. Participants were asked to rate the acceptability of the six meal items and the overall meal acceptability on a 9‐point hedonic scale. Multiple imputation was used to fill in any missing data, and multiple linear regression analysis was then performed. Through this study, a model that predicts the overall meal acceptability of vegetarian diets based on individual meal category components was developed with an *R*‐squared value of .7033, suggesting that around 70.33% of the variation in overall meal acceptability could be explained by the nine food category predictors. The findings also suggested that the most influential food item on overall meal acceptability was the main dish followed by the side dish. For every unit increase in the acceptability score of the main dish on a 9‐point hedonic scale, the overall meal acceptability increased by 0.324 points. The positive findings would be beneficial to food service providers in designing a vegetarian meal with high meal acceptability. By focusing on optimizing the acceptability of these two key categories, menu planners can effectively elevate customers' satisfaction of their meals.

## Graphical abstract

To promote the shift toward plant‐rich diets and ultimately improve the sustainability of food systems, meal acceptability testing is crucial in planning vegetarian meals. A model that predicts the overall meal acceptability of vegetarian diets based on individual meal components was developed with an *R*‐squared value of .7033. The findings suggested that the most influential food item on overall meal acceptability was the main dish followed by the side dish.

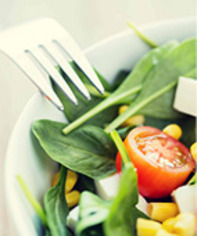

## 1 INTRODUCTION

With increasing concerns about health, animal welfare, and the environment, changes in dietary patterns are emerging, as evidenced by the gradual shift toward plant‐based diets. The percentage of US vegan consumers, for example, grew from 1% to 6% between 2014 and 2017 (Forgrieve, 2021). Plant‐based food dollar sales in the US grew three times faster than overall food sales reaching $7.4 billion in 2021 (The Good Food Institute, 2018). Plant‐based milk dollar sales in the US reached $2.6 billion in 2021, whereas those for plant‐based meat increased by 74% surpassing the growth rate of conventional meat sales (The Good Food Institute, 2018). That market is not limited to vegetarian or vegan consumers, as reflected in the increasing number of mainstream consumers who purchase such products. In the US, for example, 98% of consumers who purchase plant‐based meat also buy conventional meats (The Good Food Institute, 2018). In 2018, 16% of food products launched in the UK, that is, one in six food products, had a vegan ingredient claim (Askew, 2019). Plant‐based diets are dietary patterns that put more weight on foods derived from plant sources while lowering consumption or excluding animal products, such as meat, poultry, fish, eggs, and dairy products. The most common subsets of plant‐based diets include: (1) Vegan diet—omits all animal products, including meat, dairy, fish, eggs, and usually honey; (2) Lacto‐vegetarian diet—excludes meat, fish, poultry, and eggs, but includes dairy products, such as milk, cheese, yoghurt, and butter; (3) Lacto‐ovo‐vegetarian diet—includes eggs and dairy, but not meat or fish; (4) Ovo‐vegetarian diet—excludes meat, poultry, seafood, and dairy products, but allows eggs; (5) Pesco‐vegetarian diet—includes fish, dairy, and eggs, but not meat; and (6) Flexitarian semi‐vegetarian diet—primarily vegetarian but includes meat, dairy, eggs, poultry, and fish occasionally or in small quantities (World Health Organization, 2021).

Literature on the health benefits of adopting plant‐based diets is extensive. Bye et al. (2021), for example, reported that the odds of developing type 2 diabetes decreased by 61.9% among vegan members, 51.4% among semi‐vegetarian members, and 38.2% among lacto‐ovo‐vegetarian members compared to non‐vegetarian members. Blanco Mejia et al. (2019) performed a meta‐analysis of 43 controlled trials identified by the U.S. Food and Drug Administration (FDA) to determine soy protein's effect on total cholesterol in adults compared with other types of protein. Consuming around 25 g of soy protein daily reduced total cholesterol by 2.8% (Blanco Mejia et al., 2019). Another study conducted by Kim et al. (2018) found a 5% lower risk of all‐cause mortality with a 10‐point unit increase in the healthful overall plant‐based diet index. Adopting plant‐based diets also has the potential to reduce the environmental impacts associated with high consumption of animal‐sourced foods (Godfray et al., 2018; Springmann et al., 2016; World Health Organization, 2021). Shifting toward plant‐based diets could reduce greenhouse gas emissions by 17%–29% as well as decrease land, water, and fertilizer use (British Nutrition Foundation, 2019).

Conventionally, research on plant‐based diets focuses mainly on the nutritional value of said diets. However, few studies have investigated the real‐world performance of various strategies to increase plant‐rich diets for health promotion and sustainability reasons. Bianchi et al. (2018) evaluated the effectiveness of various interventions aimed at altering physical environments to reduce meat consumption. Reducing meat portion sizes and providing alternative meat options were shown to have the greatest impact on decreasing meat consumption (Bianchi et al., 2018). Another study conducted by Vellinga et al. (2022) found that increasing supermarket meat prices by 30% and promoting environmental awareness successfully reduced meat consumption within a virtual supermarket setting.

Additionally, fewer studies have evaluated the acceptability of such diets among populations. This evidence would be crucial to appropriately plan vegetarian meals with high consumer satisfaction that would in turn help promote this dietary pattern and ultimately improve the sustainability of food systems. Food acceptability is usually measured using a 9‐point hedonic test administered to a large group of panelists and is influenced by several factors that can be associated with the type of food, the individual consuming that said food, and the environment in which that food is being consumed (Meiselman, 2007). Such factors include sensory properties of the food, previous experience with that food, an individual's culture and physiological status, and contextual factors among others (Meiselman, 2007).

While palatability is dependent on the sensory properties of the food has a clear link with food acceptability, other factors may not have such obvious associations with it. Regarding the previous experience factor, for example, Wardle et al. (2003) suggested that repeatedly exposing children to the taste of an unfamiliar food item significantly increased their liking for that food. Studies undertaken by King et al. (2004) on the effects of contextual factors on meal acceptability suggested that serving foods as a meal in a natural eating environment and giving individuals the choice of which foods to eat increase meal acceptability. Meal acceptability ratings have also been shown to vary depending on where the meal is served. For example, Meiselman et al. (2000) found that acceptability ratings were lower in institutional settings in comparison with restaurant settings. Edwards et al. (2003) obtained similar results when testing the acceptability of the same prepared food in 10 various locations categorized by either an institutional or a restaurant setting. Such a variation in acceptability may be attributed to differences in consumer expectations of the products in these different locations.

Typically, foods are consumed as a meal but generally evaluated individually, and hence testing the acceptability of single foods on their own does not adequately predict their acceptability in the context of a meal. Assessing whole meal acceptability provides richer information on meal satisfaction than a simple collection of acceptability ratings for meal components. Meal components have varying effects on the overall acceptability (King et al., 2004). For example, Turner and Collison (1988) assessed the acceptability of a typical “dinner” meal based on the acceptability of starter, entrée, potato, vegetable, or dessert components and found that the entrée item had the highest impact on overall acceptability. Hedderly and Meiselman (1995) investigated meal acceptability in a free‐choice environment and found that the most influential was also the entrée. Elbushra and Mathews (1991) considered the main dish as the most influential component by including a question about its taste in a meal acceptability questionnaire without mentioning other meal components. Studies have so far investigated a standardized Western regular type of diet and meals; thus, it would be useful to investigate which meal component has the largest impact on meal acceptability in non‐standard diets, such as a vegetarian diet. Food categories are not as clear‐cut in a vegetarian diet as in the case of a regular diet. Consequently, investigating meal acceptability in such diets may provide more insight into the nature of the mechanisms responsible for determining meal acceptability. The present work aims to develop a model for predicting overall meal acceptability based on individual meal category components in a vegetarian diet.

## 2 MATERIALS AND METHODS

### 2.1 Samples

In the present work, 41 vegetarian meals were developed by a team at Cornell University, Ithaca, New York. Each meal consisted of various food categories, including main dish, side dish, appetizer, salad, dessert, soup, bread, beverage, and a control food. The control food is a staple food with average acceptability that is regularly consumed. Following preference testing, three control foods were used in the present study, namely commercial whole wheat bread (Wegmans'® or Thomas' Sahara®) and crackers (Stoned Wheat Thins®). A total of 220 developed foods were divided into appropriate food categories based on culinary considerations, such as the specific role of a certain food in a meal and the similarity of certain dishes to recipes consumed in regular American diets. For example, “Sloppy Joe Tempeh,” a dish similar to regular “Sloppy Joe,” was considered a main dish. Examples of some meal items used in this study included carrots with tofu peanut miso dip as an appetizer, spinach, tomato, and garlic dish as a side dish, carrot soup as a soup item, greens with mustard vinaigrette as a salad item, roasted vegetable sandwich as a main dish, soy bread as a bread item, tofu custard pie as a dessert, and chocolate soymilk as a beverage item among others (see appendix for full food list). The database of individual foods used in this study including their ingredients, recipes, processing information, and nutritional analyses had been developed in a previous study published by the authors (Hunter et al., 1998). Samples were served in sensory cups, each containing 20 g of food and given in a randomized sequence to the panelists for tasting.

### 2.2 Subjects

A total of 94 panelists (47 males and 47 females; 30–50 years of age) were recruited from Cornell University staff. Participants filled in a consent form that provided information on the study, its aim, and what they would be tasked with. They were also informed that their participation is voluntary and that they may withdraw from the survey at any point in time without giving a reason. The study was approved by the Cornell University Committee on Human Subjects. Two groups of panelists (20–24 subjects per group) were asked to participate in four major taste‐testing periods that took place over a year and a half. In the event a regular subject was absent from a tasting session, an alternate subject filled in for him or her. Twenty‐four regular panelists participated in periods one and two, 16 of whom participated again in period three and 10 in period four. Panelists were selected based on the following inclusion criteria: (a) participants should be omnivores who consumed meat on a regular basis to ensure that no bias to vegetarian meals occurs; (b) their weight had not fluctuated by no more than ±5 lbs within the last year; (c) they were not on medications; (d) they were not following any special diet; and (e) they did not have a dislike for a large number of foods. Dislike was measured using a 9‐point hedonic scale, whereby a score of 5 or lower indicated dislike. Participants who disliked no more than 10%–15% of foods were selected similar to what was done by Meiselman and Waterman (1978) to ensure that participants were generally open to a wide variety of foods.

### 2.3 Procedure

The 41 developed vegetarian meals were assessed by two groups of 20–24 non‐vegetarian panelists over four taste‐testing periods. Each meal consisted of six menu items to ensure that the collection of tested foods resembled a typical meal, and the combination of menu items was randomized. Thus, in some cases, one or more food categories, out of the total nine food categories, could be missing from a meal given to a panelist. For example, a panelist could have received two side dishes without dessert, instead of one side dish and a dessert. However, six main dishes or six desserts, for example, were never given to a panelist together as a meal. In each tasting session, participants were asked to rate the acceptability of individual foods as well as the acceptability of the meal (set of all foods) on a 9‐point hedonic scale. The presentation order of the various food categories in a taste session was randomized to eliminate any influence food item sequence could have on meal acceptability, and the participants were not told which food category each served item belonged to.

A collection of 120 food items were tested with the first group of panelists in period one, and again in period two with the second group of panelists. Commercial whole wheat bread (Wegmans'®) and crackers (Stoned Wheat Thins®) were used as control foods in periods one and two. A similar scenario took place in periods three and four with a new set of 90 foods, and an additional control food (whole wheat pita bread, Sahara®) was added.

### 2.4 Data management

Results obtained from the four testing periods were compiled into one complete dataset. It should be noted that in case two foods of the same menu category were present in one meal, their average acceptability rating represented that said category. As a result, the number of menu categories fluctuated between four and six in some cases. Data organization steps resulted in a dataset with meal acceptability as the outcome variable and the individual acceptability of the nine food categories (main dish, side dish, appetizer, dessert, salad, bread, soup, beverage, and control food) as predictor variables.

### 2.5 Statistical analyses

The design of the present meal acceptability study was determined by the requirements of a larger project rather than being designed on its own (Hunter et al., 1998), which resulted in missing data. Consequently, multiple imputation on STATA® (14.2 version) was used to account for these missing data. This method uses a regression model to predict the missing variable(s) from a present one. It is considered a rigorous method to deal with missing data and analyzing sampling variability of imputed values and is widely recognized and used for dealing with missing data in statistical analysis (Buuren, 2018; Little & Rubin, 2019; Schafer, 1999).

The “control food” variable was used as a predictor in the imputation regression model. Since the latter food category also contained missing information, mean imputation using an average “control food” acceptability of 7.3 on a 9‐point hedonic scale was first performed to fill in its missing data points. Once values for “control food” were established, multiple imputation using five imputation sets was done on the remaining variables and it was then followed by regression analyses on the new imputed dataset. In the regression analyses, meal acceptability was used as the dependent variable, whereas the various food category variables, including a main dish, side dish, appetizer, salad, soup, bread, dessert, beverage, and a control food, were all used as independent variables. A *p* value < .05 was considered statistically significant as this value is a widely accepted threshold commonly used in statistical analysis.

A new dichotomous variable defined as “new meal acceptability” was created. This variable took on a value of zero when the overall meal acceptability score was less than or equal to five, that is, a poor acceptability score, and a value of one when that score was greater than five, that is, a high acceptability score. Logistic regression was then performed by considering the new category meal acceptability variable as the outcome variable and the nine food category variables as independent variables. Classifying meal acceptability as either poor or high streamlines the decision‐making process for menu planners, enabling them to quickly identify which meal would be well received by customers.

## 3 RESULTS AND DISCUSSION

The acceptability ratings of the various food categories and the overall meal acceptability, summarized in Table 1, suggest that the various foods' scores were toward the positive side (i.e., ≥5 on the 9‐point hedonic scale) with the lowest average score for “beverage” (5.75 ± 1.94). The “main dish” achieved the highest acceptability score (6.98 ± 1.405), indicating that it was well‐liked, though it displayed some variability in preferences. The “side dish” category followed closely with a mean acceptability score of 6.88 ± 1.32, indicating strong acceptability with less variability compared to the “main dish.” The “desert” and “salad” meal components were also well‐received, achieving mean acceptability scores of 6.91 ± 1.68 and 6.94 ± 1.44, respectively. The “appetizer,” “soup,” and “bread” components exhibited slightly lower mean scores and considerable variability in their acceptability.

**TABLE 1:** Acceptability scores for meal components and overall meal acceptability.

Variable	Mean	Std. dev.	Min	Max
Main dish	6.98	1.41	1	9
Side dish	6.88	1.32	1	9
Appetizer	6.66	1.58	1	9
Dessert	6.91	1.68	1	9
Salad	6.94	1.44	2	9
Soup	6.62	1.66	1	9
Bread	6.64	1.61	1	9
Beverage	5.75	1.94	1	9
Meal acceptability	6.82	1.33	1	9

### 3.1 Multiple imputation

The resulting imputation variance analysis of each of the nine food category items is summarized in Table 2. The within variance (*V*_W_), defined as the variance within each imputed dataset for a specific variable, is simply the average of the sampling variances obtained from each of the five imputed datasets (StataCorp, 2013). To estimate the within variance for the “main dish” variable, one could average all of the squared standard errors for the “main dish” variable across the five imputed datasets. Thus, the within variance estimates the variability that would have been expected had there been no missing data. Conversely, the between variance (*V*_B_), defined as the variance across the imputed datasets for a specific variable, measures how much the estimates of this variable change from one imputation to another (StataCorp, 2013). The between variance reflects the uncertainty that results from the prediction process done to fill in missing data in the multiple imputation procedure. The total variance then becomes the sum of the within‐ and between variance as well as an additional source of sampling variance, calculated by dividing the between variance with the error associated with the average coefficient estimates (StataCorp, 2013).

**TABLE 2:** Imputation variance of the nine food category items.

Variable	*V*_W_	*V*_B_	*V*_T_	FMI
Main dish	0.000157	0.000483	0.000736	0.832
Side dish	0.000193	0.003141	0.003962	0.965
Appetizer	0.000131	0.001592	0.002042	0.953
Dessert	0.000104	0.002303	0.002867	0.974
Salad	0.000146	0.001952	0.002488	0.957
Soup	0.000116	0.001312	0.00169	0.950
Bread	0.000125	0.001606	0.002052	0.956
Beverage	0.000085	0.002853	0.003509	0.983
Control	0.000254	0.00229	0.003002	0.938

Abbreviations: FMI, fraction of missing information; *V*_B_, between variance; *V*_T_, total variance; *V*_W_, within variance.

As for the fraction of missing information (FMI), this parameter represents the proportion of the total sampling variance resulting from missing data (StataCorp, 2013). Thus, an FMI value of 0.953 for the “appetizer” variable, for example, suggested that 95.3% of the total sampling variance could be attributed to missing data. Obtained FMI values for the various food category items were large ranging between 83.2% in the case of the “main dish” variable, which had the least percentage of missing data (around 16%), and 98.3% in the case of the “beverage” variable, which had the highest percentage of missing data (around 90%).

### 3.2 Regression model

Following multiple imputation, regression analysis was performed on the imputed dataset, whereby the various food category variables were used as predictor variables to estimate the overall meal acceptability. This resulted in the following regression model:

(1) $$\begin{array}{c} \text{Meal acceptability}=-3.10+0.324\;\text{main dish}+0.27\;\text{side dish}+0.177\;\text{salad}+\\ 0.168\;\text{bread}+0.148\;\text{appetizer}+0.142\;\text{soup}+0.119\;\text{beverage}+0.113\;\text{dessert}+0.01\;\text{control}. \end{array}$$

The above model had an R‐squared value of .7033, which means that 70.33% of the variation in the overall meal acceptability could be explained by the nine food category variables: main dish, side dish, salad, bread, appetizer, soup, beverage, dessert, and control food. The average relative efficiency of the obtained regression model was found to be 84.2%, indicating that the selected model is an efficient model for estimating overall meal acceptability. The coefficients, standard errors, and 95% confidence intervals for the predictor variables in the regression model are summarized in Table 3.

**TABLE 3:** Regression model for meal acceptability dataset.

Variable	Coefficient	Standard error	[95% confidence interval]
Main dish	0.324***	0.027	[0.259 to 0.390]
Side dish	0.270*	0.063	[0.098 to 0.441]
Appetizer	0.148*	0.045	[0.027 to 0.269]
Dessert	0.113	0.054	[−0.036 to 0.261]
Salad	0.177*	0.050	[0.043 to 0.312]
Soup	0.142*	0.041	[0.033 to 0.252]
Bread	0.168*	0.045	[0.046 to 0.289]
Beverage	0.119	0.059	[−0.048 to 0.286]
Control	0.010	0.055	[−0.134 to 0.154]
Constant	−3.100*	0.427	[−4.168 to −2.033]

* *p* < .05.

*** *p* < .001.

The “main dish” variable was the most influential variable in estimating the overall meal acceptability. For every unit increase in the acceptability of the main dish, the overall meal acceptability would increase by 0.324 points on the 9‐point hedonic scale while adjusting for the remaining food category variables. The “main dish” variable was a significant predictor of overall meal acceptability with a *p*‐value < .001. Other significant predictors of overall meal acceptability also included the “side dish,” “appetizer,” “salad,” “soup,” and “bread” variables (*p*‐value < .05). The “main dish” variable was followed in importance by the “side dish” variable. That is, for every unit increase in the acceptability of the side dish, the overall meal acceptability would increase by 0.27 points on the 9‐point hedonic scale while adjusting for the other food category variables.

The obtained results were in accordance with results found in the literature. Earlier work also suggested that the main dish in a meal contributed the most to overall meal acceptability (Meiselman, 2007). Hedderly and Meiselman (1995), for example, assessed the acceptability of university cafeteria meals freely selected by students in the UK. Their results suggested that the main dish had a varying influence on meal acceptability based on the type of the main dish as well as the type of the meal.

### 3.3 Logistic regression

Results from the logistic regression model were expressed as odds ratio (OR) with 95% confidence intervals (CI). Table 4 summarizes the odds ratio as well as the 95% CI corresponding to each food category variable outlined above. Results suggested that the likelihood of having a high meal acceptability score would increase when individual food component acceptability increased, and this result was statistically significant in the case of all food component variables apart from the control food component. The main dish and side dish were also the most influential components on meal acceptability. Results revealed that those who had high main dish acceptability had higher odds of having high meal acceptability score than those who had low main dish acceptability (OR = 2.33, *p* < .05). Moreover, the odds that a high meal acceptability score would be obtained when the side dish acceptability was high was 2.05 times the odds of achieving such a score when the side dish acceptability was low while controlling for the remaining food component variables.

**TABLE 4:** Logistic model parameters for meal acceptability dataset.

Variable	Odds ratio	Std. err.	[95% confidence interval]
Main dish	2.333***	0.083	[2.176–2.501]
Side dish	2.049***	0.082	[1.896–2.216]
Appetizer	1.599***	0.053	[1.498–1.707]
Dessert	1.379***	0.039	[1.305–1.456]
Salad	1.747***	0.061	[1.632–1.871]
Soup	1.532***	0.048	[1.441–1.629]
Bread	1.776***	0.059	[1.663–1.896]
Beverage	1.394***	0.037	[1.324–1.468]
Control	0.933	0.040	[0.857–1.015]

*** *p* < .001.

## 4 CONCLUSION

In conclusion, the main dish was found to be the most influential item on overall meal acceptability, even in such a vegetarian meal setting. The side dish was second in importance in meal acceptability after the main dish. These conclusions were in accordance with previous meal acceptability studies, despite the fact that these studies considered non‐vegetarian meals. Future work will investigate whether the impact of culturally acquired beliefs and certain nutritional parameters is related to vegetarian meal acceptability, in addition to the impact of individual food category acceptability and meal acceptability on food consumption in a multiple food setting.

As previously mentioned, the design of the present study was determined by the requirements of a larger study rather than being designed on its own. Consequently, the number of food categories given to a panelist was not uniform across the various meals given to the panelists. All panelists received six food items, but one may have two appetizers with no soup dish, for example, and another might have two soup dishes with no appetizer. Additionally, sociodemographic factors that might affect participants' meal acceptability ratings were not explored in this study. Future work will be based on balancing food categories in the meal evaluation sessions, whereby panelists will have a full regular meal instead of tasting the sample food components and sociodemographic factors will be considered when developing the meal acceptability model. This might shed more light on the role of each food category in overall meal acceptability. Moreover, this work paved the way for a 30‐day diet study where the foods were consumed ad libitum (Olabi et al., 2015).

## AUTHOR CONTRIBUTIONS

**Caroline Rajeh:** Formal analysis (equal); methodology (equal); software (equal); writing – original draft (equal). **Jean B. Hunter:** Conceptualization (equal); supervision (equal). **David A. Levitsky:** Conceptualization (equal); supervision (equal). **Myra Zeineddine:** Investigation (equal). **Samer A. Kharroubi:** Formal analysis (equal); software (equal); validation (equal). **Ammar Olabi:** Conceptualization (equal); investigation (equal).

## CONFLICT OF INTEREST STATEMENT

The authors declare no potential conflict of interest.

## Data Availability

The data that support the findings of this study are available on request from the corresponding author.
